# Television decreases intra-operative hypertensive events in cataract surgery: a randomized clinical trial

**DOI:** 10.1007/s00417-026-07140-4

**Published:** 2026-02-25

**Authors:** Tiffany Cheng, Ricky Paramo, Michael Curley, Sruthi Kodali, Amanda Zong, Anne Barmettler

**Affiliations:** 1 Department of Ophthalmology and Visual Sciences, Montefiore Medical Center, Albert Einstein College of Medicine, Bronx, NY USA; 2 Department of Ophthalmology, Jefferson Health, Wills Eye Hospital, Philadelphia, PA USA; 3 Department of Ophthalmology, Jamaica Hospital Medical Center, Queens, NY USA; 4 Department of Ophthalmology, Boston Medical Center, Boston, MA USA; 5 Montefiore Medical Center, Albert Einstein College of Medicine, Department of Ophthalmology, 3332 Rochambeau Ave, 3rd Floor, Bronx, NY 10467 USA

**Keywords:** Cataract surgery, Music, Television, Intra-operative hypertension, Anxiety

## Abstract

**Purpose:**

To compare the effects of television (TV) vs. music vs. control on hypertensive events and anxiety in cataract surgery.

**Methods:**

A prospective, single-masked, randomized controlled trial of 476 adult cataract surgery patients at a surgical center in New York. Patients were randomized to either listen to classical music, view a home design and renovation program, or a control group. Blood pressure, heart rate, and anxiety levels (VAS-A) were measured before and 20 minutes post-intervention. Intra-operative hypertensive events (IOEs) were recorded.

**Results:**

Of 650 enrolled patients, 476 (27% exclusion) completed the study and a per-protocol analysis was used. In first-time cataract surgeries, the TV group had significantly fewer IOEs than controls (0 (0%) vs. 9 (10.3%), difference 10.3%; 95% CI, 3.9% to 16.7%; *p* = 0.003). Music did not show a significant IOE reduction vs. control (6 (6.4%) vs. 9 (10.3%), difference − 4.0%; 95% CI, -12.0% to 4.1%; *p* = 0.334). TV trended toward fewer IOEs than music but did not reach significance (0 (6.4%) vs. 6 (0.0%), difference 6.4%; 95% CI, 1.4% to 11.3%; *p* = 0.030, adjusted *p* = 0.025). Decreased VAS-A was reported between music vs. control (0.88 vs. 0.17, difference 0.71; 95% CI, 0.39 to 1.03), TV vs. control (0.84 vs. 0.17, difference 0.67; 95% CI, 0.35 to 1.02), music vs. TV (0.88 vs. 0.84, difference 0.04; 95% CI, -0.32 to 0.37).

**Conclusions:**

Viewing TV before surgery was associated with fewer IOEs in first-time cataract surgery patients. Music and TV were associated with lower pre-operative anxiety and might contribute to decreased IOEs that can be explored in future studies.

**Trial registration:**

NCT06356324 (04/2024).

**Supplementary Information:**

The online version contains supplementary material available at 10.1007/s00417-026-07140-4.

## Key Messages

*****What is known***:**

- While music has been shown to decrease anxiety and peri-operative hypertensive events during cataract surgery, the effects of audiovisual interventions, such as television, have not been evaluated in a randomized study.

*****What is new***:**

- This study is the first to establish that viewing television, specifically home improvement shows, directly prior to cataract surgery in the pre-operative setting helps reduce anxiety and the incidence of intra-operative hypertensive events.
- This study also confirmed that listening to classical music decreases peri-operative anxiety.

**Supplementary Information:**

The online version contains supplementary material available at 10.1007/s00417-026-07140-4.

## Introduction

Music has been demonstrated to alleviate anxiety associated with ophthalmic surgery, which is generally performed under minimal sedation. Reducing anxiety is advantageous for patients as it can lower vital signs, decrease unintentional movement during surgery, and enhance overall mental well-being. Intra-operative anxiety and fear can lead to unintended movements, which can cause serious surgical complications during critical phases of the procedure. Research indicates that reducing anxiety can help minimize spikes in blood pressure, respiration, and heart rate [1]. This, in turn, decreases the risk of intra- and peri-ocular bleeding [2–4]. In cataract surgery, listening to calming music prior to or during surgery significantly reduces anxiety, blood pressure, and tachycardia [5, 6], while listening to calming music during surgery can reduce salivary alpha-amylase, a marker of stress-related autonomic activity [7]. While music has been established to decrease peri-operative anxiety and related markers, there is a paucity of research examining the effects of audiovisual interventions in ophthalmic surgery. Studies involving ophthalmic surgeries are limited to one study, where watching patient information videos before cataract surgery reduced pre-operative anxiety; however, this study was not randomized, and many patients may prefer not to view educational content immediately before surgery [8]. Given that cataract surgery is the most frequently performed surgery worldwide, and with increased frequency in recent years, there is an increased interest to optimize operating conditions to minimize surgical complications and enhance patient experience [9]. To date, the effects of non-medical videos on more objective measures, such as hypertension, have not been explored in the ophthalmic setting. To date, this is the first randomized controlled trial to investigate the impact of television vs. music vs. control in the immediate preoperative period on anxiety and intra-operative hypertensive events (IOEs) in patients undergoing cataract surgery.

## Methods

This was a prospective, single-masked, randomized controlled trial with three arms: music, television (TV), and control. This study was Institutional Review Board approved and Health Insurance Portability and Accountability Act compliant, adhering to the Declaration of Helsinki and registered with ClinicalTrials.gov (NCT06356324; ClinicalTrials.gov; https://clinicaltrials.gov*).* The ophthalmic surgeon, anesthesiologist, and operating room staff were masked. Patients and researchers were masked to allocation until the administration of interventions.

Inclusion criteria involved adult cataract surgery patients (first-eye or second-eye) recruited from November 2021 through January 2024 at an ambulatory surgery center in New York, NY. Surgeries were limited to cataract extractions to minimize variability in surgery type and length. Participants were excluded if they had severe hearing loss, psychiatric disorders, dementia, or any condition precluding the ability to obtain informed consent. The following was collected directly prior to the intervention: demographics, first- or second-time cataract surgery, baseline systolic (SBP) and diastolic blood pressure (DBP), heart rate (HR), and anxiety levels (measured on a scale from 0 to 10) using the Visual Analogue Scale for Anxiety (VAS-A), a previously validated tool to measure subjective anxiety levels [10]. Uncontrolled hypertension was defined as SBP greater than 140 mmHg and/or DBP greater than 90 mmHg.

Participants were randomized into one of three arms: music, TV, or control. Blocking randomization included a 1:1:1 ratio with blocks of 3, 6, and 9 using an online random number generator, Research Randomizer [11]. In all three arms, the intervention occurred in the patient’s pre-operative room for 20 minutes. The lights were dimmed, and the curtains were drawn. BP, HR, and VAS-A levels were re-measured at the end of the 20-minute intervention in the same pre-operative room prior to being taken to the operating room. Of note, the research staff was not masked to group assignment but were instructed not to disclose additional information regarding the goals of the study to limit bias. Participants in the music arm listened to a set playlist of classical music at a comfortable audio level on computer speakers. Speakers were chosen over headphones because the latter would require additional equipment and cleaning. Set music was chosen for standardization and to avoid disruption of the hospital environment. In the TV arm, participants watched and listened to a home design and renovation video clip on a computer screen at a comfortable audio level. The screen was adjusted to the optimal height and distance for the participant. Home improvement videos, like HGTV, were chosen as they tend to be universally understood and neutral in content. In the control arm, participants’ room were similarly dimmed as in the intervention groups but were instructed to wait and relax and received no additional intervention.

The primary outcome was the incidence of at least one IOE during cataract surgery. This was defined as SBP greater than 160 mmHg and/or DBP greater than 100 mmHg combined with a HR greater than 85 beats per minute. Secondary outcomes included the change in SBP, DBP, HR, and VAS-A scores after the 20-minute intervention.

Monitored anesthesia care was provided with pre-medication of intravenous midazolam (1–2 mg) and fentanyl (50–100 mcg) for sedation and pain control. Additional administration of sedatives or pain-control agents was at the discretion of the anesthesiologist. Routine cataract extraction was then performed with phacoemulsification and endocapsular lens implantation. BP and HR were monitored every 3 to 5 minutes. The number of IOEs and procedure length were recorded.

### Statistical analyses

As per prior methodologies, a sample size of 155 participants per arm was required for 80% power at a *p* < 0.05 significance level to detect a 20% change in IOEs between the control and intervention arms. The rates assumed for intervention and control group was a 20% frequency of at least 1 IOE based on previous studies [6]. Anticipating a distracting intervention, such as music or TV, would reduce the frequency of IOEs by 50%, a sample size of 155 per group was deemed necessary to detect the expected difference. Statistical analysis was performed using R (R Foundation for Statistical Computing, Vienna, Austria). Continuous variables, baseline blood pressure, heart rate, and anxiety levels, exhibited skewed distributions and were summarized using medians and interquartile ranges (IQR). Non-parametric methods were used for group comparisons: Mann-Whitney U or Kruskal-Wallis tests (including Dunn’s post-hoc testing). Categorical variables, IOEs, were summarized as counts and percentages and analyzed using a chi-square or Fisher’s exact test (including the Holm-Bonferroni method). Bootstrap resampling was performed to estimate the mean changes and corresponding 95% confidence intervals (CI). Primary outcomes were stratified by first- or second-time surgery in each arm. Secondary outcomes were evaluated by comparing changes in variables from pre- to post-intervention. A per-protocol analysis was used.

## Results

### Baseline characteristics

**Fig. 1:**
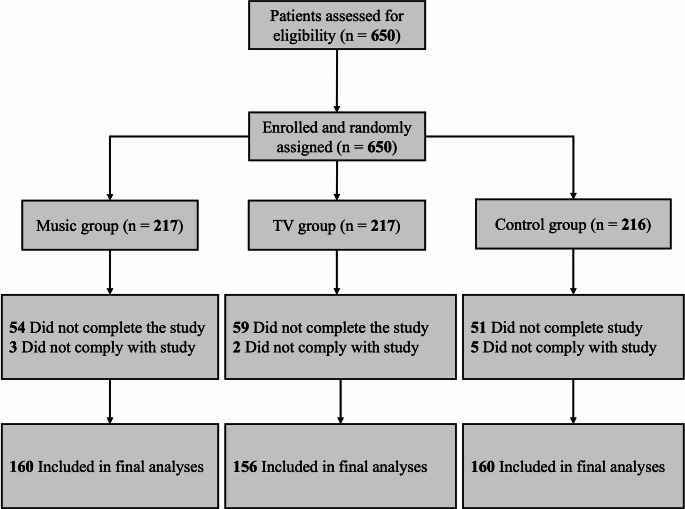
Selection process flowchart for the study

Of the 650 participants, 174 (27%) were excluded, 164 due to inadequate time to complete the intervention as the patient was taken to the operating room, and 10 for poor compliance. The remaining 476 participants had been randomized: 160 to the control arm, 156 to the TV arm, and 160 to the music arm (Fig. 1).

Of the 476 participants, 294 (61.8%) were female with a mean (SD) age of 70.1 ± 9.4 years. At baseline, 257 participants had well-controlled hypertension, 156 had poorly controlled hypertension, and 63 did not have a prior diagnosis of hypertension. The eligibility criteria were chosen to ensure comparable baseline characteristics, including baseline physiologic measurements. Additionally, prior cataract surgery experience was included as a prognostic variable for additional comparison. Table 1 shows baseline characteristics among the three arms with respect to age, sex, control of hypertension, and first- vs. second-time cataract surgeries (Table 1).

**Table 1:** Baseline participant characteristics

Characteristic	Total (*N* = 476)	Music (*N* = 160)	TV^a^ (*N* = 156)	Control (*N* = 160)	
Age, mean (SD)	70.1 (9.4)	70.4 (9.1)	69.3 (9.2)	70.6 (9.3)	
Sex, n (%)					
Male	182 (38.2)	52 (32.5)	60 (38.5)	70 (43.4)	
Female	294 (61.8)	108 (67.5)	96 (60.0)	90 (56.3)	
1^st^ or 2^nd^ surgery, n (%)					
1^st^	265 (55.6)	94 (58.8)	84 (53.8)	87 (47.5)	
2^nd^	211 (44.3)	66 (41.3)	72 (46.2)	73 (45.6)	
HTN^b^ control at baseline, n (%)					
Yes	257 (55)	83 (51.9)	90 (57.7)	84 (52.5)	
No	156 (34.7)	56 (35.0)	44 (28.2)	56 (35.0)	
NA^c^	63 (10.3)	21 (13.1)	22 (14.1)	20 (12.5)	

^a^*TV* television; ^b^*HTN* hypertension; ^c^*NA* not applicable, indicating no prior history of HTN

## Intra-operative hypertensive events

**Fig. 2:**
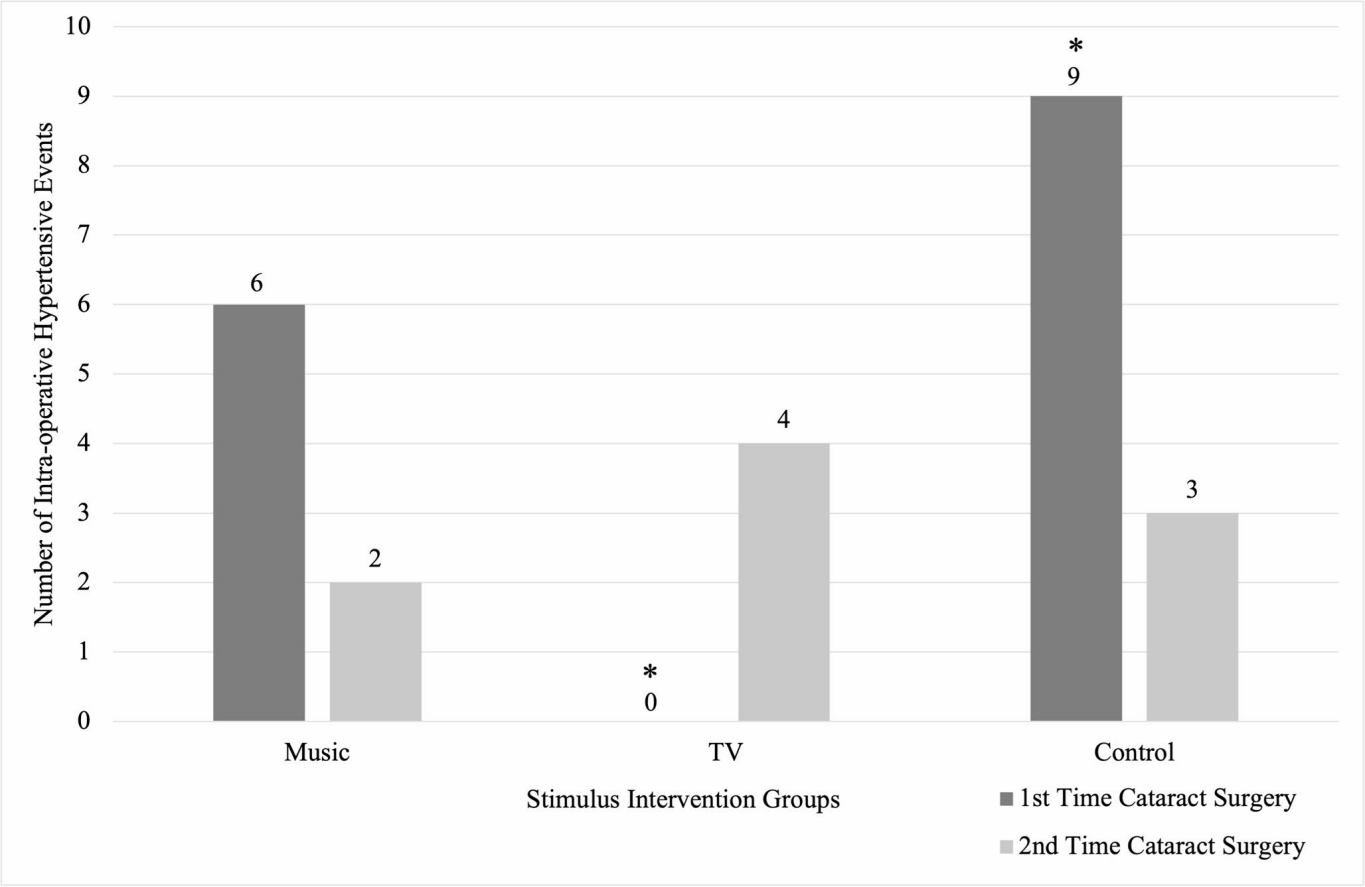
In first-time cataract surgery patients, there was a statistically significant decrease in the number of intra-operative hypertensive events recorded in patients watching TV compared to the control arm. TV = television. *Statistically significant

In first-time cataract surgery patients, there was a statistically significant difference in the number of IOEs among the music (6 (6.4%)), TV (0 (0.0%)), and control (9 (10.3%)) groups (*p* = 0.005) (Fig. 2).

IOEs were more frequently associated with higher preoperative SBP (IOE median SBP: 144 vs. non-IOE median SBP: 132; difference 12 min; 95% CI, 19.5 to 3.5 min; *p* = 0.001). The median duration of surgical cases was significantly longer in patients with IOEs than in those without IOEs (23.5 vs. 14 min; difference 9.5 min; 95% CI, 8 to 18 min; *p* < 0.001). The overall duration of surgery for each group was as follows: music (14 min), TV (13 min), and control (15 min). Among the groups for surgery length, only TV vs. control had a significant difference in surgical length (13 vs. 15 min, *p* = 0.018). In first-time cataract surgery patients, adjusting the initial analysis for multiple comparisons for specific pairwise analysis (music vs. control; TV vs. control), the incidence of IOEs in the TV group was significantly lower than in the control group (0 (0.0%) vs. 9 (10.3%); difference 10.3%; 95% confidence interval (CI), 3.9% to 16.7%; *p* = 0.003) via the Holm-Bonferroni method with adjusted significance level of 0.025 for chi-square or Fisher’s exact tests. However, there was no statistically significant difference in the pairwise analysis between IOEs for the following:

- First-time cataract surgery music and control groups (6 (6.4%) vs. 9 (10.3%), difference − 4.0%; 95% CI, -12.0% to 4.1%; *p* = 0.334).
- First-time cataract surgery music and TV groups (6 (6.4%) vs. 0 (0.0%), difference 6.4%; 95% CI, 1.4% to 11.3%; *p* = 0.030).
- Second-time cataract surgery TV and control groups (4 (5.6%) vs. 3 (4.1%), difference 1.4%; 95% CI, -5.5% to 8.4%; *p* = 0.719).
- Second-time cataract surgery music and control groups (2 (3.0%) vs. 3 (4.1%), difference − 1.1%; 95% CI, -7.2% to 5.1%; *p* = 1.000).
- Second-time cataract surgery music and TV groups (2 (3.0%) vs. 4 (5.6%), difference − 2.5%; 95% CI, -9.2% to 4.2%; *p* = 0.682).

When comparing IOEs in all three groups, there was no statistically significant difference among first- and second-time cataract surgery patients (15 (5.7%) vs. 9 (4.3%); difference 1.4%; 95% CI, -2.5% to 5.3%, *p* = 0.490). In patients undergoing second-time cataract surgery, no differences were observed in all three groups (*p* = 0.839) (Table 2).

**Table 2:** Incidence of intra-operative hypertensive events

First-time surgery (*N* = 265)	Music (*N* = 94)	TV^a^ (*N* = 84)	Control (*N* = 87)	*p* value
Intra-operative hypertensive events, n (%)	6 (6.4)	0 (0)	9 (10.3)	0.005*
Second-time surgery (*N* = 211)	Music (*N* = 66)	TV (*N* = 72)	Control (*N* = 73)	*p* value
Intra-operative hypertensive events, n (%)	2 (3.0)	4 (5.6)	3 (4.1)	0.839

^a^*TV* television. *Statistically significant

## Other outcome measures

After the 20-minute intervention, a post-hoc analysis (Dunn’s test) of VAS-A demonstrated a statistically significant difference between music vs. control groups (*p* < 0.001) and TV vs. control groups (*p* < 0.001). Mean differences for both music and TV were associated with decreased VAS-A between music vs. control (0.88 vs. 0.17, difference 0.71; 95% CI, 0.39 to 1.03), and TV vs. control (0.84 vs. 0.17, difference 0.67; 95% CI, 0.35 to 1.02). Mean differences of VAS-A between music vs. TV was not significant (0.88 vs. 0.84, difference 0.04; 95% CI, -0.32 to 0.37). Additionally, there were statistically significant differences between baseline music VAS-A and post-20-minute music VAS-A groups (*p* < 0.001), and statistically significant differences between baseline TV VAS-A and post-20-minute TV VAS-A groups (*p* < 0.001). However, there was no statistically significant difference in VAS-A changes in the music vs. TV arms (*p* = 0.665), baseline control VAS-A and post-20-minute control VAS-A groups (*p* = 0.070), or significant differences in median BP or HR otherwise (Table 3). Additionally, adjustments for covariates were done for baseline imbalances given the selected per-protocol data in this study (Online Resource 1).

**Table 3:** Outcome measures before and after audiovisual intervention

Measurement^a^	Median (IQR^b^)			Intra-group mean changes$\Delta$(95% CI)			*p* value
Before audiovisual intervention	Music (*N* = 160)	TV^f^ (*N* = 156)	Control (*N* = 160)	Music ∆ (95% CI)	TV ∆ (95% CI)	Control ∆ (95% CI)	
Systolic BP^c^, mmHg	133 (121-147)	130 (118-142)	134 (122-146)	-3.3 (-5.4 to -1.3)	-1.4 (-3.9 to 1.3)	-2 (-4.1 to 0.1)	0.563
Diastolic BP, mmHg	73 (67-82)	74 (65-80)	75 (66-82)	-1.8 (-3.5 to -0.1)	-1.1 (-2.7 to 0.4)	-1.2 (-2.6 to 0.1)	0.322
HR^d^, bpm	73 (64-82)	70 (63-83)	74 (65-82)	-5.5 (-6.4 to -4.6)	-4.7 (-5.9 to -3.6)	-5.7 (-6.9 to -4.5)	0.505
VAS-A^e^	3 (0-5)	3 (0-5)	2 (0-5)	-0.9 (-1.1 to -0.6)	-0.8 (-1.1 to -0.6)	-0.17 (-0.4 to 0.1)	< 0.001*
After audiovisual intervention				Between-Group Differences$\Delta$(95% CI)			
	Music (*N* = 160)	TV (*N* = 156)	Control (*N* = 160)	∆ Control - Music	∆ Control - TV	∆ TV - Music	
Systolic BP, mmHg	130 (117-141)	128 (116-140)	130.5 (121-144)	-1.3 (-4.2 to 1.6)	0.6 (-2.3 to 4.2)	-2.0 (-5 to 1.2)	
Diastolic BP, mmHg	70 (63-79)	72 (63.5-79)	74 (66-80)	-0.6 (-2.6 to 1.7)	0.1 (-2.0 to 2.0)	-0.7 (-2.8 to 1.7)	
HR, bpm	67.5 (58-76)	66.5 (59-75)	67.5 (59-75.5)	0.2 (-1.4 to 1.6)	1.0 (-0.7 to 2.5)	-0.8 (-2.3 to 0.6)	
VAS-A	2 (0-5)	2 (0-5)	2 (0-5)	-0.7 (-1.0 to -0.4)	-0.7 (-1.0 to -0.4)	0 (-0.4 to 0.3)	

^a^Measurements of physiologic and anxiety levels before and after 20 minutes of audiovisual intervention. ^b^*IQR* interquartile ranges; ^c^*BP* blood pressure; ^d^*HR* heart rate; ^e^*VAS-A* visual analog scale for anxiety; ^f^*TV* television. ∆ = difference between subgroups. CI = confidence interval.^*^ Comparing mean differences of intra-group changes among music, TV, and control groups in VAS-A measurements yielded a *p-*value < 0.001. Specifically, VAS-A intra-group changes between music vs. control and TV vs. control were statistically significant, *p* < 0.001. Music vs. TV was not statistically significant, *p* = 0.665

## Discussion

While music can reduce anxiety and its related markers in ophthalmic surgeries, the effects of audiovisual interventions are less known [1, 5–8, 12]. To date, there are no studies in ophthalmology that examine the peri-operative physiologic effects of non-educational TV, and none that have compared TV with music. Additionally, prior studies utilized headphones, which is limiting, as it may not be feasible to supply or clean headphones for pre-operative patients. The study herein is the first randomized, controlled, single-masked study to observe the anxiolytic effects of a 20-minute exposure, without headphones, of music vs. non-educational TV vs. control in 476 cataract surgery patients. Intra-operative hypertensive events (IOEs) were the primary outcomes. Exposure to a 20-minute classical music or a home improvement TV series intervention significantly reduced subjective pre-operative anxiety levels.

First-time cataract surgery patients had significantly fewer intra-operative hypertensive events after watching home improvement TV for 20 minutes compared to controls before surgery. Of note, TV also had fewer IOEs compared to music. Although this difference was not significant (*p* = 0.030, with adjusted significance of *p* = 0.025 for multiple comparisons), it was approaching significance and suggests a larger sample size could potentially identify TV as a more effective measure compared to music in preventing IOEs. These findings suggest that audiovisual interventions may have sustained therapeutic effects on anxiety and physiologic markers beyond the pre-operative period. Intriguingly, patients in this study had a significant reduction in preoperative anxiety (VAS-A), but no significant immediate reduction in physiological markers of anxiety (BP or HR). Nevertheless, as observed in this study, patients exposed to TV preoperatively had beneficial physiologic outcomes in terms of fewer IOEs. This phenomenon was similarly observed in the study by Guerrier et al. (2021), where they found no significant immediate reduction in physiologic markers of anxiety (BP or HR) preoperatively, albeit with music in their case vs. TV or music in this study. Regardless, findings of their study and this study herein suggest the effects of a distracting stimulus in the immediate preoperative setting likely are preventing an undesired physiologic outcome rather than producing an immediate measurable physiologic change. Such phenomena might be related to other physiologic variables (e.g., cortisol levels) that are not immediately reflected in other physiologic markers of anxiety (BP or HR). Future studies can explore the relationship between other physiologic markers and subsequent pharmacologic sedation as related to intraoperative events such as hypertensive episodes. Similar findings were seen in prior studies, but they used videos with medical content, required an hour of TV viewing, required patients to come in the day prior to surgery to watch the video, or didn’t measure physiologic outcomes like blood pressure or heart rate [8, 12–14]. Application of these prior protocols was limited, as patients may not wish to watch detailed scientific videos prior to surgery, and it is not logistically practical to make patients come early or on the day prior to surgery to watch a video for an hour. On the other hand, the results of the authors’ study demonstrate a practical method to decrease anxiety and potentially IOEs via a shorter course of exposure on the same day of surgery, with the use of non-educational video content, and without headphones.

This study also identified an association between surgical length and the incidence of IOEs, with longer surgeries correlating with a higher incidence of IOEs. The increased surgical length may reflect an increase in surgical complications related to increased IOEs. If blood pressure is high and there is more intraocular bleeding, this can lengthen a surgery. Or perhaps the patient ascertains there is a surgical complication, becomes more anxious, and this results in elevated blood pressure. The association could also reflect that longer surgeries increase the probability of an IOE. Future research could help clarify this relationship.

Interestingly, our cohort of patients had low anxiety levels at baseline, which may account for the small differences seen in the VAS-A scores and physiologic measures of anxiety (BP and HR). Albeit there were statistically significant intra-individual changes seen between baseline VAS-A and post-intervention VAS-A levels for both music and TV groups. This suggests a measurable reduction of anxiety preoperatively that may have positive lasting effects, as the intervention groups had fewer IOEs compared to the control group in this study. This may be due to patients’ experience with prior, more invasive surgeries, as many had undergone lengthier surgeries, such as coronary bypass, bilateral hip replacements, etc., preparing them for subsequent less-invasive surgeries. Prior patient experiences with surgery may also be the reason behind the lack of difference in IOEs in second-time cataract surgery patients.

In terms of comparing the clinical relevance of our outcome measures to the literature, one prior validation study identified a VAS-A score of approximately 4.6/10 as a threshold for clinically significant preoperative anxiety [10]. Additionally, another study identified a two-point change in VAS as a threshold for clinical significance, extrapolated from minimal clinically important difference (MCID) assessments of VAS-measured pain [15]. Finally, prior studies with similar surgical populations reported between-group VAS-A differences (music vs. control) of approximately 1.5 points (95% CI, 1.0 to 2.1), which were associated with meaningful downstream outcomes such as reduced intraoperative anxiolytic administration [6]. Using these benchmarks in our per-protocol analysis, it is unlikely that the adjusted difference between music and control, which was 0 (95% CI, 0 to 2), would exceed MCID, although smaller clinical effects cannot be definitively excluded.

Similarly, a formal MCID for IOEs has not been established. The literature instead defines these events with the following thresholds: SBP > 160 mmHg or SBP > 160 and/or DBP > 100 mmHg combined with HR > 85 bpm [6, 16], which delineate clinically concerning intraoperative states rather than thresholds for meaningful risk reduction. In this context, clinical relevance was assessed by examining the magnitude and confidence limits of absolute risk differences. In the herein per-protocol analysis, the difference in IOE incidence between music and control was 4% (95% CI, -12.0% to 4.1%), suggesting the absence of a clinically meaningful reduction. In contrast, the difference between TV and control was 10.3% (95% CI, 3.9% to 16.7%), indicating a clinically relevant reduction in IOEs given the potential impact of these events on intraoperative management and surgical workflow.

Limitations to this study include the subjective nature of the anxiety rating scale, although the Visual Analogue Scale is a previously validated method to assess anxiety. Second, the variable nature of surgery and surgeons precludes the complete standardization of the surgeries. Third, due to the surgical center’s efficiency, there was a relatively large number of excluded patients, who were taken to the operating room prior to completing the full intervention, which may have contributed to a selection bias. The enrollment of patients during their preoperative time was balanced with the permission of the surgical center, and patients were not held longer than regularly needed to not interfere with the surgical center’s workflow. Future research can investigate the minimal time needed (e.g., 10 vs. 20 min) for music vs. TV to have similar outcomes in terms of anxiety and IOEs to accommodate the workflow of busy surgical centers. Notably, patients who did not complete the study in its entirety had their enrollment data discarded. As such, this study serves as a per-protocol study, and future trials can explore the outcomes of all participants with variable exposure times to music or TV. Lastly, vital signs were used as proxies for anxiety, and this can be limiting as they can be influenced by factors such as pain.

This prospective, randomized controlled study of 476 cataract surgery patients is the first to demonstrate the benefits of music or TV interventions in reducing pre-operative anxiety without the need for headphones. Additionally, 20 minutes of non-medical TV decreased the incidence of intra-operative hypertension in first-time cataract surgery patients. Providing classical music and home improvement TV in the pre-operative area can be an inexpensive yet effective way to decrease subjective markers of anxiety, reduce the incidence of intra-operative hypertensive events, and ultimately improve patient experience.

## Supplementary Information

Below is the link to the electronic supplementary material.

Supplementary Material 1 (DOCX 32.4 KB)

Supplementary Material 2 (PDF 63.7 KB)

## References

[1] Dahshan D, Kuzbel J, Verma V (2021) A role for music in cataract surgery: a systematic review. Int Ophthalmol 41(12):4209–4215. 10.1007/s10792-021-01986-9 34312781 10.1007/s10792-021-01986-9

[2] Méndez-Ulrich JL, Sanz A, Feliu-Soler A, Álvarez M, Borràs X (2018) Could white coat ocular hypertension affect to the accuracy of the diagnosis of glaucoma? Relationships between anxiety and intraocular pressure in a simulated clinical setting. Appl Psychophysiol Biofeedback 43(1):49–56. 10.1007/s10484-017-9385-x 29119282 10.1007/s10484-017-9385-x

[3] Brody S, Erb C, Veit R, Rau H (1999) Intraocular pressure changes: the influence of psychological stress and the Valsalva maneuver. Biol Psychol 51(1):43–57. 10.1016/s0301-0511(99)00012-5 10579420 10.1016/s0301-0511(99)00012-5

[4] Speaker MG, Guerriero PN, Met JA, Coad CT, Berger A, Marmor M (1991) A case-control study of risk factors for intraoperative suprachoroidal expulsive hemorrhage. Ophthalmology 98(2):202–210. 10.1016/s0161-6420(91)32316-9 2008278 10.1016/s0161-6420(91)32316-9

[5] Wiwatwongwana D, Vichitvejpaisal P, Thaikruea L, Klaphajone J, Tantong A, Wiwatwongwana A (2016) The effect of music with and without binaural beat audio on operative anxiety in patients undergoing cataract surgery: a randomized controlled trial. Eye (Lond) 30(11):1407–1414. 10.1038/eye.2016.160 27740618 10.1038/eye.2016.160 PMC5108018

[6] Guerrier G, Abdoul H, Jilet L, Rothschild PR, Baillard C (2021) Efficacy of a web App-Based music intervention during cataract surgery: A randomized clinical trial. JAMA Ophthalmol 139(9):1007–1013. 10.1001/jamaophthalmol.2021.2767 34323929 10.1001/jamaophthalmol.2021.2767 PMC8323047

[7] Musa A, Ng QX, Wai YZ, Iqbal T (2021) Effect of slow tempo music on markers of anxiety during cataract surgery: randomized control trial. Taiwan J Ophthalmol 12(1):74–81. 10.4103/tjo.tjo_10_21 35399979 10.4103/tjo.tjo_10_21 PMC8988978

[8] Ahmed KJ, Pilling JD, Ahmed K, Buchan J (2019) Effect of a patient-information video on the preoperative anxiety levels of cataract surgery patients. J Cataract Refract Surg 45(4):475–479. 10.1016/j.jcrs.2018.11.011 30709627 10.1016/j.jcrs.2018.11.011

[9] Rossi T, Romano MR, Iannetta D, Romano V, Gualdi L, D’Agostino I, Ripandelli G (2021) Cataract surgery practice patterns worldwide: a survey. BMJ Open Ophthalmol 6(1):e000464. 10.1136/bmjophth-2020-000464 33501377 10.1136/bmjophth-2020-000464 PMC7812090

[10] Facco E, Stellini E, Bacci C, Manani G, Pavan C, Cavallin F, Zanette G (2013) Validation of visual analogue scale for anxiety (VAS-A) in preanesthesia evaluation. Minerva Anestesiol 79(12):1389–1395 23860442

[11] Urbaniak GC, Plous S (2013) Research R. Updated 2015. https://randomizer.org/ Accessed 01 Nov 2021.

[12] Chow CH, Van Lieshout RJ, Schmidt LA, Dobson KG, Buckley N (2016) Systematic review: audiovisual interventions for reducing preoperative anxiety in children undergoing elective surgery. J Pediatr Psychol 41(2):182–203. 10.1093/jpepsy/jsv094 26476281 10.1093/jpepsy/jsv094 PMC4884908

[13] Friedman SB, Badere B, Fitzpatrick S (1992) The effects of television viewing on preoperative anxiety. J Post Anesth Nurs 7(4):243–250 1494991

[14] Doering S, Katzlberger F, Rumpold G, Roessler S, Hofstoetter B, Schatz DS, Behensky H, Krismer M, Luz G, Innerhofer P, Benzer H, Saria A, Schuessler G (2000) Videotape Preparation of patients before hip replacement surgery reduces stress. Psychosom Med 62(3):365–373. 10.1097/00006842-200005000-00010 10845350 10.1097/00006842-200005000-00010

[15] Dziadzko M, Mazard T, Bonhomme M, Raffin M, Pradat P, Forcione J, Minjard R, Auburn F (2022) Preoperative anxiety in the surgical transfer and waiting area: A Cross-Sectional mixed method study. J Clin Med 11(9):2668. 10.3390/jcm11092668 35566793 10.3390/jcm11092668 PMC9100941

[16] Bos EME, Tol JTM, de Boer FC, Schenk J, Hermanns H, Eberl S, Veelo DP (2024) Differences in the incidence of hypotension and hypertension between sexes during Non-Cardiac surgery: A systematic review and Meta-Analysis. J Clin Med 13(3):666. 10.3390/jcm13030666 38337360 10.3390/jcm13030666 PMC10856734

