# Supplementary material for: Television decreases intra-operative hypertensive events in cataract surgery: a randomized clinical trial
Source: Graefes Arch Clin Exp Ophthalmol. 2026 Feb 25;264(6):1671–8. doi: 10.1007/s00417-026-07140-4 (PMC13197346; doi:10.1007/s00417-026-07140-4)
Supplement: Supplementary file 2 — Supplementary Material 2 (PDF 63.7 KB) [file 417_2026_7140_MOESM2_ESM.pdf]

Online Resource 1 Adjusted intra-group outcomes (ANCOVA) and change scores

**Post-intervention means**				
**Group**	Adjusted SBP[b], mean (95% CI)	Adjusted DBP[c], mean (95% CI)	Change score* HR[d], mean (95% CI)	Change score VAS-A[e], mean (95% CI)
Music	130 (128 – 132)	71.8 (70.4 – 73.3)	-5.5 (-6.6 to -4.4)	-0.9 (-1.1 to -0.6)
TV[a]	131 (129 – 133)	72.4 (70.9 – 73.9)	-4.7 (-5.9 to -3.6)	-0.8 (-1.1 to -0.6)
Control	131 (129 – 134)	72.6 (71.1 – 74.1)	-5.7 (-6.8 to -4.6)	-0.2 (-0.4 to 0.1)
**Pairwise adjusted mean differences**				
**Comparison**	Adjusted SBP, mean difference (95% CI)	Adjusted DBP, mean difference (95% CI)	Change score HR, mean difference (95% CI)	Change score VAS-A, mean difference (95% CI)
Music vs Control	1.6 (-1.4 to 4.5)	0.7 (-1.3 to 2.8)	-0.2 (-2.0 to 1.6)	0.7 (0.4 to 1.0)
TV vs Control	0.2 (-2.8 to 3.2)	0.2 (-1.9 to 2.3)	-1.0 (-2.8 to 0.9)	0.7 (0.3 to 1.0)
Music vs TV	1.4 (-1.7 to 4.4)	0.6 (-1.5 to 2.7)	0.8 (-1.1 to 2.6)	0 (-0.3 to 0.4)

[a]TV = television; [b]SBP = systolic blood pressure; [c]DBP = diastolic blood pressure; [d]HR = heart rate; [e]VAS-A = Visual Analog Scale for Anxiety; CI = confidence interval. *For HR and VAS-A scores, a significant group and baseline interaction was observed, indicating that the relationship between baseline and post-intervention HR and VAS-A differed across groups. Therefore, standard ANCOVA was not used for these measurements. Instead, intra-individual change scores were analyzed and compared between groups.
